# Revisiting Diabetic Myonecrosis: A Case Report Highlighting Diagnostic and Management Challenges

**DOI:** 10.7759/cureus.109268

**Published:** 2026-05-20

**Authors:** Doaa M Sabir, Marwa Elaziz, Sidra M Nayyar, Hadeel H Mohamed Elhassan, Dharmesh Shukla

**Affiliations:** 1 Emergency Medicine, Hamad Medical Corporation, Doha, QAT

**Keywords:** cellulitis differentials, diabetes complications, diabetic myonecrosis, long standing diabetes complications, poorly controlled diabetes complications

## Abstract

A 41-year-old man with a history of uncontrolled type 2 diabetes mellitus presented with acute thigh pain and fever. He had tense muscle compartments, skin discoloration, and erythema with elevated inflammatory markers on blood labs. Ultrasonography revealed grossly edematous muscles, and culture showed growth of streptococcal dygalactae. Biopsy revealed histiocytic infiltrates with interstitial edema of muscle fibers. The patient was treated with surgical exploration, fluids, and broad-spectrum antibiotics, which led to complete recovery. This case highlights the potential development of diabetic myonecrosis owing to suboptimal blood glucose control. As well it demonstrates the pathophysiology, presentations, and management approach of the disease.

## Introduction

Diabetes mellitus (DM) is a condition characterized by persistent hyperglycemia due to inadequate or abnormal insulin secretion, differences in insulin resistance, or a combination of these two mechanisms [1].

Approximately 830 million individuals worldwide have diabetes, with the majority living in low- and middle-income nations. The proportion of individuals with diabetes, as well as those who are untreated, has continuously increased to 14% of adults older than 18 years in 2022, compared with 7% in 1990. In 2008, it was found that almost 59% of adults with diabetes aged 30 years and older did not take any medication to manage their condition [2].

Although DM is associated with the typical complications of neuropathy, nephropathy, retinopathy, and cardiovascular disease, it may also cause numerous musculoskeletal diseases. Though uncommon, these musculoskeletal complications are clinically significant and include such conditions as limited joint mobility, tendon pathology, and, in rare situations, diabetic myonecrosis.

A microvascular complication of diabetes known as diabetic myonecrosis or diabetic muscle infarction (DMI) is observed in patients with poorly controlled DM of long duration. It was first observed by Angervall and Stener in 1965, who documented tumor-like lesions in diabetic patients with ischemic muscle degeneration. Since its original description, fewer than 200 cases have been reported in the literature [3].

The definite pathogenesis of DMI remains unknown, although theories suggest that microvascular sequelae of diabetes are involved, including muscular injury as a result of hypoxia-reperfusion disturbances, occlusion due to atherosclerosis, vasculitis complicated by thrombosis, and atheroembolism of the small vessels. Some authors have also suggested that diabetes causes disturbances in the coagulation-fibrinolysis system, resulting in hypercoagulability and vascular endothelial damage, which may represent a possible mechanism for this condition [4].

Diabetic myonecrosis is a spontaneous process, and most of the time, there is no history of trauma or infection, which usually leads to misdiagnosis as abscesses, tumors, or myositis. Affected patients usually complain of pain and swelling of the affected muscles, often in the proximal lower extremities, although involvement may also be distal or bilateral. DMI should be considered by clinicians managing patients with poorly controlled diabetes because it often presents with tenderness and swelling in the limbs, mimicking deep vein thrombosis, particularly when venous ultrasound findings are negative. Radiological assessment, especially MRI, plays a vital role because it is a noninvasive tool for identifying typical muscle edema and necrosis, assessing the extent of involvement, and helping distinguish DMI from other conditions, whereas CT and ultrasonography are used as additional modalities [5-8].

Our case highlights the educational value of recognizing diabetic myonecrosis as a rare but important differential diagnosis in patients with poorly controlled diabetes presenting with acute limb pain, particularly when clinical and radiological features mimic necrotizing soft tissue infection.

## Case presentation

**Figure 1:**
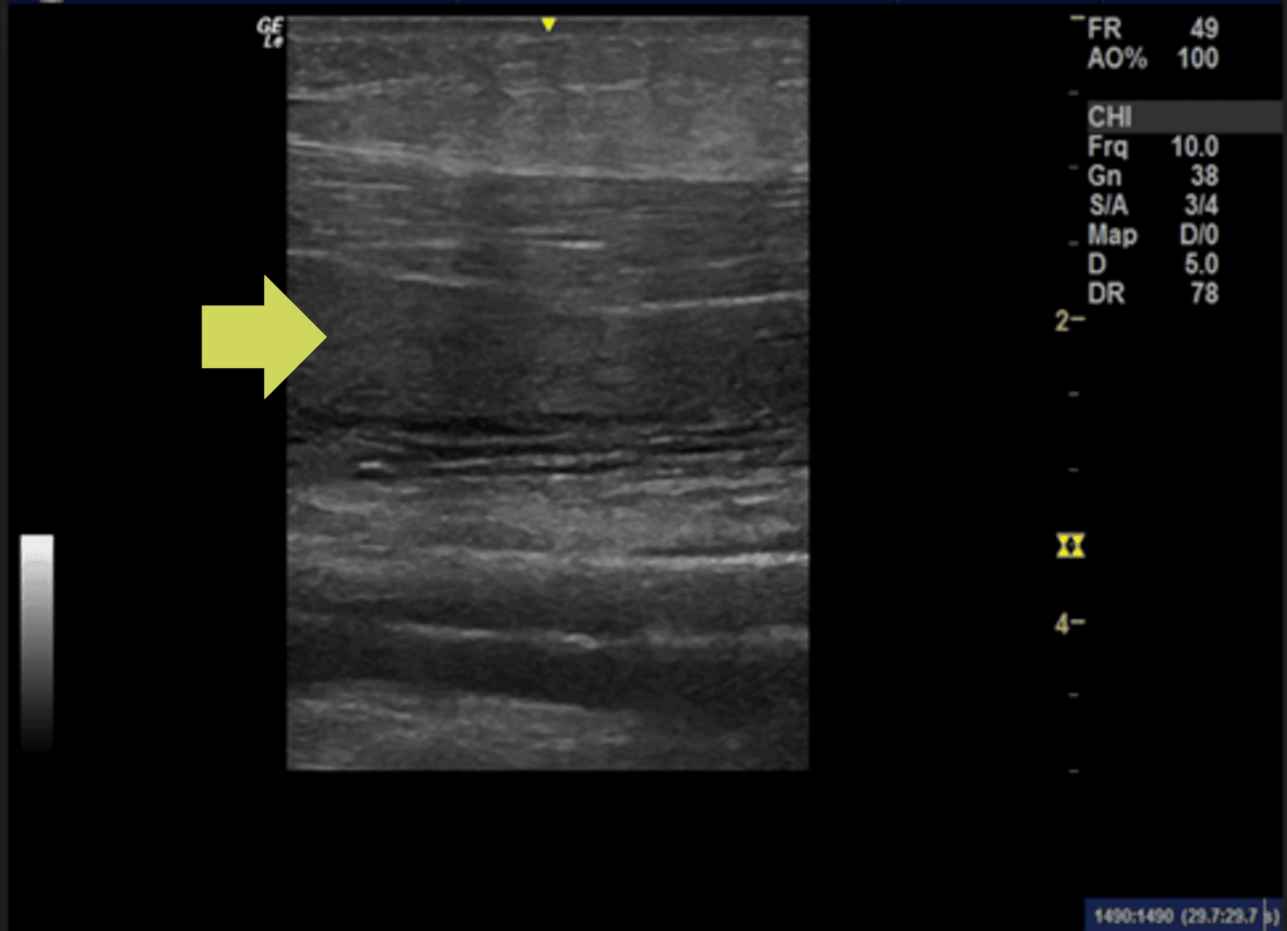
POCUS findings: the right thigh showing muscle edema with loss of striae. POCUS: point-of-care ultrasound.

**Figure 2:**
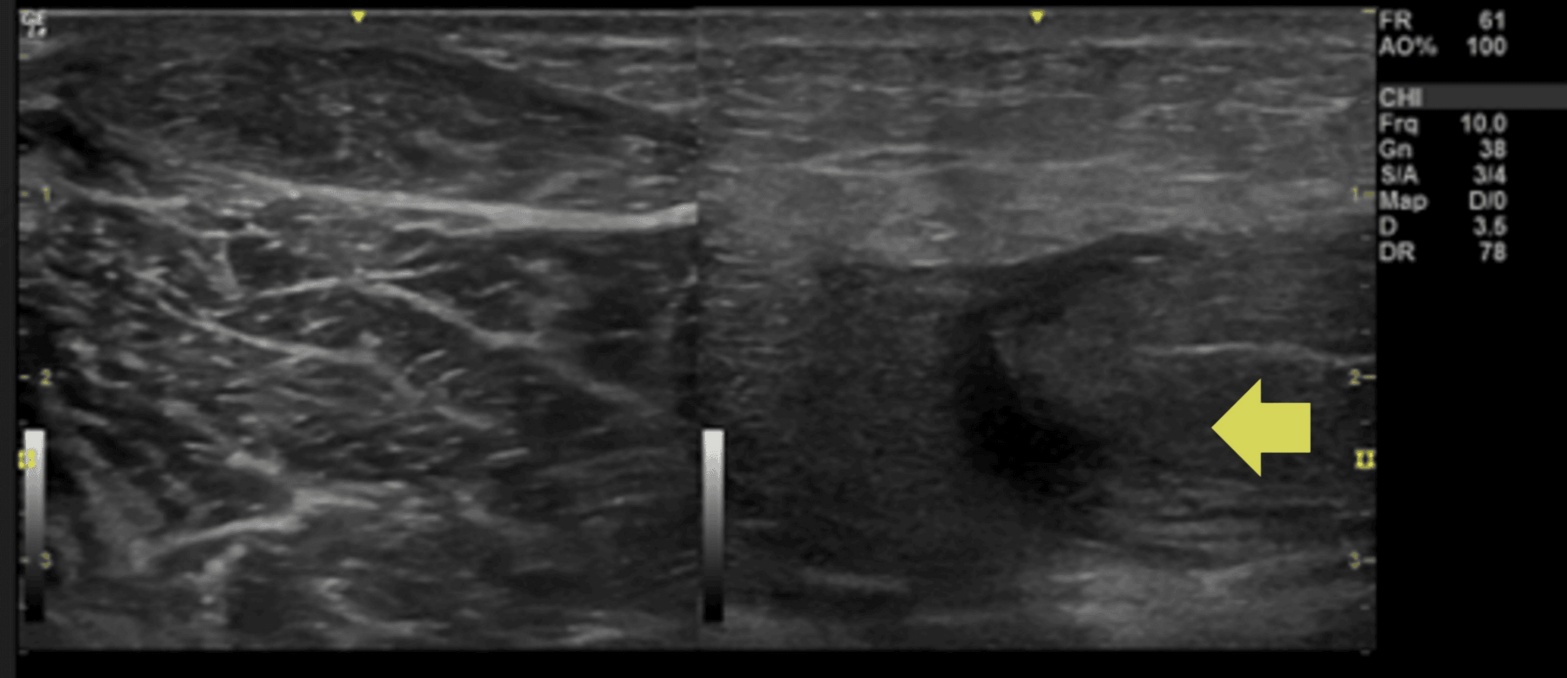
POCUS findings: the left normal thigh muscles and right edematous thigh muscles. POCUS: point-of-care ultrasound.

**Figure 3:**
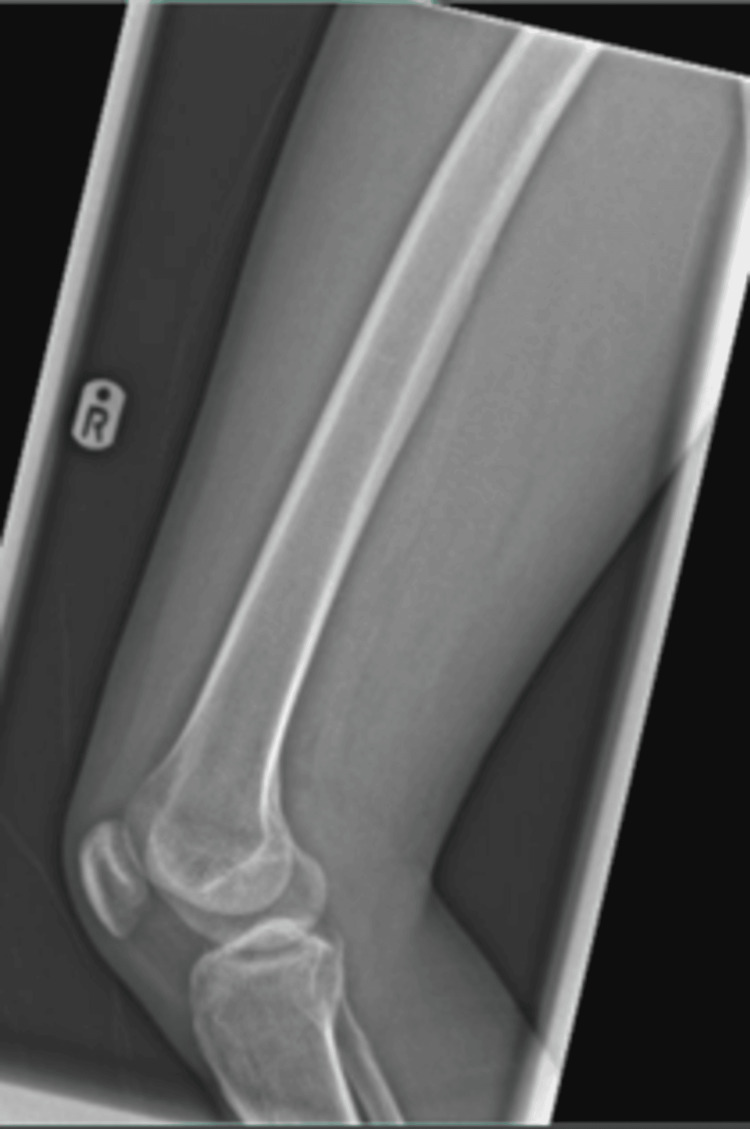
X-ray of the femur.

**Figure 4:**
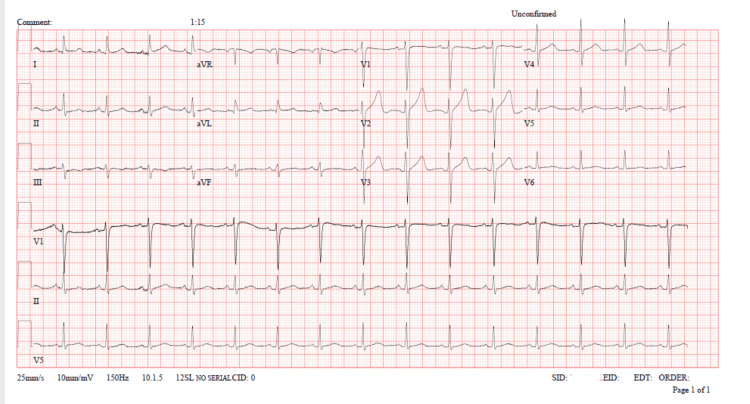
ECG: normal sinus rhythm.

This is a case of a 41-year-old man who presented to the emergency department with complaints of acute right thigh pain for four days, associated with subjective fever for two days. This was the first time he had developed such symptoms, and there was no associated recent travel, trauma, nausea, or vomiting. His medical history included type 2 diabetes over the last 15 years; however, he was not compliant with his oral antidiabetic medications. His vital signs were as follows: blood pressure 120/77 mmHg, temperature 37 °C, heart rate 108 beats/minute, respiratory rate 19 breaths/minute, and oxygen saturation 99% on room air. Local examination revealed warmth, severe tenderness to touch, swelling over the anteromedial aspect of the right thigh, erythema, bluish-brown skin discoloration, and a mildly tense thigh. In addition, there were multiple indurations with no crepitus, and the neurovasculature was intact. The remainder of his systemic examination, including the genital examination, was unremarkable. Given the history and examination findings, essential differential diagnoses for this presentation included necrotizing fasciitis (NF) of the thigh, cellulitis, and deep vein thrombosis (DVT). We initiated management with intravenous normal saline fluids, which were later switched to lactated Ringer's solution. Broad-spectrum antibiotics, such as piperacillin-tazobactam and clindamycin, along with insulin and salbutamol for hyperkalemia, were also initiated. The workup was directed toward the above-mentioned differentials. Laboratory investigations revealed hyperglycemia (random blood sugar 22.1 mmol/L), elevated white blood cell count (WBC = 23.9 × 10³/µL), elevated C-reactive protein (CRP = 290 mg/L), hyperkalemia (K = 5.8 mmol/L), and elevated renal function markers (urea 9.4 mmol/L, creatinine 118 µmol/L) (Table 1), in addition to a normal anion gap and the absence of acidosis on venous blood gas (VBG) analysis. In terms of radiological investigations, bedside point-of-care ultrasound (POCUS) was performed, which illustrated the presence of generalized muscular edema without evidence of subcutaneous air collection, while official radiological ultrasound did not reveal any evidence of DVT or abscess collection (Figures 1, 2). The femur radiograph did not exhibit gas formation around the bone (Figure 3). The electrocardiogram (ECG) showed a normal sinus rhythm (Figure 4). 

**Table 1:** Laboratory results.

Laboratory Test	Before Treatment	After Treatment	Normal Value
White blood cell count (WBCs)	23.9 × 10^3^/mL	9.2 × 10^3^/mL	4-10 × 10^3^/mL
Hemoglobin (Hb)	11.4 g/dL	10.1 g/dL	L
Platelets	393 × 10^3^/mL	493 × 10^3^/mL	150-410 × 10^3^/mL
Absolute neutrophil count (ANC)	21 × 10^3^/mL	6.4 × 10^3^/mL	2-7 × 10^3^/mL
C-reactive protein (CRP)	290.6 mg/L	44.8 mg/L	0.0-5 mg/L
Beta-hydroxybutyrate	3 mmol/L	<0.10 mmol/L	0.03-0.3 mmol/L
Creatinine	118 mmol/L	99 mmol/L	62-106 mmol/L
Blood urea nitrogen (BUN)	9.4 mmol/L	9.8 mmol/L	2.5-7.8 mmol/L
Creatinine kinase (CK)	870 U/L	681 U/L	39-308 U/L
Myoglobin	397 ng/mL	238 ng/mL	28-72 ng/mL
Aspartate aminotransferase (AST)	24 U/L	22 U/L	0-40 U/L
Alanine aminotransferase (ALT)	20 U/L	28 U/L	0-41 U/L
Alkaline phosphatase (ALP)	191 U/L	175 U/l	40-129 U/L
Total bilirubin	9 mmol/L	3 mmol/L	0-21 mmol/L
Hba1c	10.2%	8.4%	<5.7%
Potassium	5.8 mmol/L	5.2 mmol/L	3.5-5.3 mmol/L
Glucose	22.1 mmol/L	6.4 mmol/L	3.3-5.5 mmol/L

In view of our concerns regarding the potential for NF, the General Surgery (GS) team was consulted and immediately took the patient to the operating theater (OT) for exploration on the same day under general anesthesia. The operative findings included grossly edematous subcutaneous tissue with tense fascial compartments. The muscles appeared swollen and hyperemic, yet contractile and viable, with no evidence of frank necrosis, purulence, or dishwater fluid. No fascial planes were disrupted, and no foul odor was appreciated. The tissue biopsy report was as follows: “Histologic sections demonstrate diffuse lymphohistiocytic infiltrates with interstitial edema affecting striated muscle fibers, in keeping with acute myositis. The absence of myofiber necrosis, neutrophilic microabscesses, or vascular thrombosis distinguishes this from necrotizing fasciitis,” confirming the diagnosis of diabetic myositis/myonecrosis.

Outcome and follow-up

A multidisciplinary team approach was applied in the patient’s care, although throughout his clinical stay, the patient remained admitted under the care of the surgery team. The endocrine team was also involved in the follow-up of his case for a total of 13 days of hospital stay, and he was later discharged in good condition and in a recovered state.

During hospitalization, management strategies included strict glycemic control, wound care, analgesics, and empirical broad-spectrum antibiotics (piperacillin/tazobactam). On the third day of admission, the superficial wound culture grew *Streptococcus dysgalactiae*; subsequently, antibiotics were changed to intravenous amoxicillin-clavulanic acid for a total duration of 14 days. However, given the negative blood cultures and lack of operative or histological evidence of deep infection, this finding was interpreted as superficial infection or colonization rather than invasive muscular infection. On the 10th day of admission, the patient underwent secondary exploration with wound closure. During hospitalization, his renal function fluctuated but normalized before discharge, without the need for dialysis. During his follow-up visit, he was found to be doing well, with significant wound improvement.

## Discussion

DMI, also known as diabetic myonecrosis or diabetic myositis, is an uncommon complication of poorly controlled, long-standing DM and is frequently misdiagnosed as cellulitis or polymyositis. So far, fewer than 200 cases have been reported since its first description in 1965 [8,9]. A systematic review has shown that, among the reported cases of DMN, women were more affected (61.5% of all cases), patients with type 1 diabetes were predominant (59% of all cases), and the condition was frequently observed in patients diagnosed with long-standing DM, with a mean duration of 14.3 years [8-11].

The pathophysiology of DMI remains unclear, although it is believed to occur due to complex microvascular ischemia, endothelial damage, and inflammatory responses. Microvascular damage decreases blood circulation and results in tissue ischemia, which subsequently triggers an inflammatory cascade leading to muscle necrosis. This damage is further enhanced by reperfusion injury, which increases oxygen radicals, depletes nitric oxide availability, and increases the release of inflammatory mediators, leading to edema and increased compartment pressure. Imaging examinations have revealed expansion of muscular arterioles, and peripheral vascular disease can cause greater impairment of perfusion. The existence of an additional coagulation-immune mechanism is suggested by hypercoagulability or the presence of antiphospholipid antibodies, which are more frequent in type 1 diabetes. Vasculitis has also been suggested, although its clinical presentation is similar to that of DMI [4].

The clinical manifestations usually include acute, rapidly progressive pain in the involved muscles. The thigh muscle group is commonly affected, with a predominance in the vastus group. Other manifestations include swelling of the affected area, tenderness, firmness, edema, and the appearance of a mass. In addition, the absence of constitutional symptoms is common, which makes the diagnosis of DMN easily overlooked because it can be challenging to differentiate it from other differential diagnoses such as deep vein thrombosis, necrotizing fasciitis, soft tissue abscess, pyomyositis, hematoma, cellulitis, and acute compartment syndrome [8,12,13].

Moreover, laboratory investigations have a limited role in aiding the diagnosis. However, leukocyte count, creatine phosphokinase, CRP, or ESR may be minimally elevated. Although the lack of correlation between muscle involvement and creatine phosphokinase levels has led to delays in seeking medical advice by approximately four weeks, our patient showed evidence of elevated WBC, CRP, and CK levels, supporting the ongoing superficial wound infective pathology [8,14-16].

Furthermore, MRI is the best modality for diagnosis, as it may show increased signaling from the affected muscle area on T2-weighted, inversion recovery, and gadolinium-enhanced images and isointense or hypointense areas on T1-weighted images. However, in this case, MRI was not performed due to urgent clinical concern for necrotizing soft tissue infection, which necessitated immediate surgical exploration. This clinical urgency precluded advanced imaging prior to operative intervention. Computed tomography is a supportive diagnostic tool that shows widespread muscular enlargement with reduced attenuation of the affected muscle, subcutaneous fascial and skin thickening, and increased attenuation of subcutaneous fat. Moreover, ultrasound scans are beneficial for demonstrating sonographic findings of DMN, such as the absence of internal motion or fluid swirling with transducer pressure, internal linear echogenic structures coursing through the lesion, and a lack of predominantly anechoic areas. Additionally, point-of-care ultrasound is another evolving tool that facilitates faster diagnosis, as the sonographic features of DMN can easily be detected. One case of DMN diagnosed via POCUS has been reported in the literature [8,10,13-15]. Our patient’s POCUS findings of muscular edema with loss of striae hinted toward the diagnosis of DMN; however, we performed it initially to explore the differential diagnosis and evaluate for abscess formation, exclude deep vein thrombosis, assess for subcutaneous emphysema, and identify abnormal muscle echotexture, despite the fact that these findings are nonspecific and may overlap with infectious myositis, cellulitis, inflammatory myopathies, and traumatic muscle injury. The ultrasound probe used was a high-frequency linear transducer (5-10 MHz). Therefore, surgical exploration was performed because necrotizing fasciitis remained a strong differential diagnosis. Although biopsy remains the gold standard modality, it is usually deferred because of the potential complications and delay in recovery [8,13,14]. Our patient underwent tissue biopsy during the operation, which showed evidence of diffuse lymphohistiocytic infiltrates with interstitial edema affecting striated muscle fibers, consistent with the diagnosis of DMN, as there was no evidence of internal muscle infection.

The management of DMN is conservative because it is a self-limiting disease. However, supportive care strategies, such as good glycemic control, bed rest, and analgesics for pain, should be implemented, and patients usually recover within a few weeks. In contrast, patients who undergo surgery have an average recovery period of around 13 weeks compared with 5.5 weeks for those treated conservatively. Despite the short-term prognosis of the condition being considered good, the long-term prognosis is poor, and recurrence occurs in 47.82% of cases [8,15,16].

## Conclusions

In conclusion, this case demonstrates the challenging and often overlooked diagnosis of diabetic myonecrosis, particularly when it resembles severe soft tissue infection. Our patient’s sudden onset of thigh pain, systemic inflammatory response, and initial ultrasound findings pointed toward necrotizing fasciitis, highlighting how easily DMN can be misdiagnosed as a surgical emergency. Therefore, this case emphasizes the importance of considering DMN in patients with long-standing poorly controlled diabetes who present with unexplained limb pain and swelling, although it remains diagnostically challenging to differentiate between the two diagnoses clinically. Considering MRI before surgical exploration may be helpful. However, more validated research is required to aid in avoiding unnecessary surgical interventions, as in our case, surgery was unavoidable. Optimizing glucose control and enhancing patient adherence are essential strategies for preventing recurrence and limiting microvascular complications.
